# Microbiome and metabolome explain the high-fat diet-induced diabetes development and diabetes resistance in Guizhou mini-pigs

**DOI:** 10.3389/fmicb.2025.1555069

**Published:** 2025-04-09

**Authors:** Yanjun Wu, Jiayuan Mo, Qianguang Wang, Jialong Li, Jia Wei, Nuo Zhang, Yuanqiu Dong, Xiang Zhu, Taofeng Lu, Sicheng Huang

**Affiliations:** ^1^The Provincial Key Miao Medicine Laboratory of Guizhou, Guizhou University of Traditional Chinese Medicine, Guiyang, China; ^2^College of Animal Science, Anhui Science and Technology University, Chuzhou, China; ^3^Anhui Engineering Technology Research Center of Pork Quality Control and Enhance, Anhui Science and Technology University, Chuzhou, China; ^4^Department of Oncology, The Affiliated Hospital of Guizhou Medical University/ Guizhou Hospital of the First Affiliated Hospital, Sun Yat-sen University, Guiyang, China; ^5^Department of Abdominal Oncology, The Affiliated Cancer Hospital of Guizhou Medical University, Guiyang, China

**Keywords:** type 2 diabetes, high-fat diet, microbiome, metabolome, Guizhou mini-pig

## Abstract

Type 2 diabetes mellitus (T2DM) is an obesity-related disease claiming substantial global mortality annually. Current animal models of T2DM remain limited, with low success rates in establishing porcine models of high-fat diet (HFD)-induced T2DM. Our experimental design employed 35 Guizhou mini-pigs to develop a T2DM model via HFD induction, aiming to identify microbial and metabolic signatures associated with disease pathogenesis and resistance. At month 10, five individuals from the control (CTR), T2DM (DM), and T2DM resistant (anti-DM) groups were slaughtered, samples were collected, and relevant indices were measured. Metagenomics, metabolomics, and 16S rRNA sequencing were performed to identify microbes and metabolites linked to T2DM progression and resistance. Key findings demonstrated anti-DM group parameters-including metabolic indices (fasting blood glucose, insulin levels, HbA1c, IVGTT), histopathology (HE-stained pancreatic/hepatic tissues), microbial profiles (structural, compositional, functional), and metabolomic signatures-occupied intermediate positions between CTR and DM groups. Network analyses revealed: (1) *Lactobacillus*, *L. amylovorus*, fingolimod, polyoxyethylene sorbitan monooleate, thiamine, and atrazine in HFD-associated networks; (2) *Limosilactobacillus reuteri*, N-oleoyl-L-serine, tolbutamide, tetradecanoyl carnitine, 3′-sulfogalactosylceramide, and guggulsterone in T2DM resistance networks; (3) *Ruminococcaceae NK4A214 group*, diethyl phthalate, zingerone, enalapril, 5-hydroxytryptophol, 2′-deoxyinosine, icariin, and emetine in T2DM progression networks. These results further clarify the role of the gut microbiota and serum metabolites in the development of T2DM in the Guizhou mini-pig model.

## 1 Introduction

Type 2 diabetes (T2DM) is a serious threat to human health (Ma, 2018). According to the International Diabetes Federation, approximately 537 million adults (10% of the global population aged 20–79 years) were living with diabetes in 2021, and this number is predicted to rise to 643 million by 2030.^1^ Currently, there are over 110 million diabetics in China (Chen X. W. et al., 2021), with a large proportion of cases attributed to nutritional factors, particularly the consumption of a high-fat diet (HFD) (Wei et al., 2021). Generally, HFD-induced obesity results from excess energy intake, causing excessive enlargement of adipocytes in white adipose tissue (Ji et al., 2023). Additionally, HFD can trigger T2DM by generating lipotoxicity, reducing energy metabolite capacity, promoting inflammation, increasing reactive oxygen species concentration, impairing autophagy, and disrupting insulin function (Heydemann, 2016). Furthermore, the relationship between HFD, obesity, and T2DM is tightly linked (Malone and Hansen, 2019; Boutari et al., 2023), for example obesity is strongly associated with the onset and severity of T2DM (Hong and Kim, 2022). Therefore, HFDs are commonly used to establish animal models of obesity and T2DM (Hu et al., 2022; Wu et al., 2018). However, the success rate of HFD-induced T2DM in Duroc pigs is only 13.3%, reportedly due to high bile concentrations of hyocholic acid in pigs (Zheng et al., 2021).

A twin study showed that gut microbes in obese individuals can induced an increase in body weight in normal individuals (Ridaura et al., 2013), and HFD promotes obesity and metabolic disease development by depleting Th17 through inducing microbial composition and function changes (Kawano et al., 2022). Reduced bacterial diversity, lower abundance of butyrate-producing bacteria (e.g., *Roseburia* and *Faecalibacterium*), and probiotic bacteris (e.g., *Bifidobacterium* and *Akkermansia*) are characteristic features in T2DM patients (Niu et al., 2022). The gut microbiota, particularly *Escherichia*/*Shigella*, *Bacteroides vulgatus*, and *Christensenella minuta*, has been linked to the development of obesity, metabolic syndrome, and T2DM (Zheng et al., 2017). Metabolites produced by gut microbiota, such as trimethylamine N-oxide and lipopolysaccharide, also play key roles in T2DM progression (Zhuang et al., 2019; Wu et al., 2023). Similarly, indole-3-propionic acid, agmatine, and tyramine-product by gut microbes-are beneficial metabolites associated with T2DM resistance (Du et al., 2022). Thus, understanding the roles of gut microbes and metabolites in T2DM is crucial for developing targeted therapeutic strategies.

The Guizhou mini-pig is an experimental miniature pig breed independently developed by Guizhou University of Traditional Chinese Medicine and is widely used in disease research (Li et al., 2016; Sun et al., 2017; Zhang Y. et al., 2019). Guizhou mini-pigs exhibit stable genetic characteristics and a well-defined genetic background. More importantly, Guizhou mini-pigs are easy to handle and surgical manipulate due to their small body size (Chen et al., 2011; Mo et al., 2022b). Additionally, their anatomical and physiological parameters are more similar to humans than those of mice (Mo et al., 2022a). Our research group successfully established a Guizhou mini-pig model of diabetic nephropathy via streptozocin injection and identified two microbial species and five metabolites associated with disease pathogenesis (Wu et al., 2024). However, the success rate of generating an HFD-induced T2DM model in Guizhou mini-pigs remains unclear, as do the mechanistic roles of gut microbiota and related metabolites.

In this study, we used Guizhou mini-pigs to successfully establish a diabetes model through HFD and obtain diabetic and diabetes-resistance individuals. Additionally, T2DM-related physiological and biochemical indicators were assessed through histopathological analysis, 16S rRNA sequencing, metagenomics, and metabolomics. Finally, microbial and metabolite networks linked to HFD-induced T2DM and disease resistance were constructed, establishing a theoretical foundation for the treatment of T2DM.

## 2 Materials and methods

### 2.1 Animals and groups

In total, 35 male Guizhou mini-pigs [aged, 6 months; mean body weight (BW): 20.02 ± 0.97 kg] were obtained from Guizhou University of Traditional Chinese Medicine (production license: SCXK[QIAN] 2021–0003; laboratory animal license: SYXK[QIAN] 2021–0005) and randomly allocated to either the control (CTR) group (*n* = 5) or the HFD group (*n* = 30). Pigs in the CTR group were fed a base formulated diet (12.95 MJ/kg digestible energy, 16% crude protein) and those in the HFD group were fed a high-fat, high-carbohydrate diet. The formulated diet was provided by Tainongqingyin Co., Ltd. (Guiyang, China), and the HFD consisted of a standard formula supplemented with 37% sucrose and 10% fat (Wu et al., 2018). All animals were housed in single-occupancy pens within a climate-controlled swine facility. The experimental procedures complied with the Chinese Regulations for the Management of Experimental Animals. The experiment lasted for 10 months (Wu et al., 2018).

### 2.2 Physiological and biochemical indices

After fasting for 16 h, BW was measured, and blood samples were collected monthly. The blood samples were centrifuged (3,000 rpm, 15 min, 4°C), and the serum was collected to measure fasting blood glucose (FBG) levels using an automatic biochemical analyzer (ADVIA2120i; Siemens AG, Munich, Germany). Serum samples collected at month 10 were used to determine insulin (INS) concentrations with a porcine INS enzyme-linked immunosorbent assay kit (Cloud-Clone Corp., Wuhan, China) and glycated hemoglobin (HbA1C) levels with a glycosylated hemoglobin quantitative detection kit (Quanzhou Ruixin Biotechnology Co., Ltd., Quanzhou, China). As described in our previous studies, the intravenous glucose tolerance test (IVGTT) was performed at month 10 (Wu et al., 2018), and pigs in the HFD groups were categorized into the DM group or anti-DM group (Wu et al., 2024). Individuals with FBG levels exceeding 7.0 mmol/L were classified as T2DM.

### 2.3 Sample collection

After the experiment, all pigs were anesthetized via ear vein injection of pentobarbital sodium and humanely sacrificed. Subsequently, the liver, pancreas, and feces were collected. The liver and pancreas were fixed within 4% paraformaldehyde, then dehydrated through a graded alcohols series, cleared in xylene, and embedded in paraffin wax. Following sectioning into 4-μm-thick slices, tissues were dewaxed using xylene, rehydrated through a descending alcohols series, and rinsed in water. Finally, sections were stained with hematoxylin and eosin (HE). Fecal samples were immediately snap-frozen in liquid nitrogen and stored at −80°C.

### 2.4 16S rRNA amplicon sequencing

Fecal microbial genomic DNA was extracted using the MagPure Stool DNA LQ Kit (Magen Biotech, Guangzhou, China) following the manufacturer’s protocol, and the 16S rRNA V3–V4 region was amplified with the primers (forward) 5′-CCTAYGGGRBGCASCAG-3′ and (reverse) 5′-GGACTACNNGGGTATCTAAT-3 (Wu et al., 2024). 16S rRNA sequencing and analysis were performed as previously described using R language with the silva_16s_v123 database (Wu et al., 2024). Parameters included a min_unique_size of 10, a minimum sequencing depth of 32,909, and Principal Component Coordinates Analysis (PCoA) based on Manhattan distances. Permutational multivariate analysis of variance (PERMANOVA) was applied to assess beta diversity significance, while permutational analysis of multivariate dispersions (PERMDISP) evaluated intergroup dispersion homogeneity. Differentially abundant microbes (DAMs) were identified via linear discriminant analysis effect size (LEfSe) with an linear discriminant analysis (LDA) score threshold >3, and significantly altered Kyoto Encyclopedia of Genes and Genomes (KEGG) pathways were determined using Structural Time Series Analyser, Modeler and Predictor (STAMP) with a significance cutoff of *p* < 0.05.

### 2.5 Metagenomic sequencing

Following the manufacturers’ protocols for the DNeasy^®^ PowerSoil^®^ Kit (Qiagen GmbH, Hilden, Germany) and the NEB Next® Ultra™ DNA Library Prep Kit for Illumina (New England Biolabs, Ipswich, MA, USA), metagenomic DNA was extracted from fecal samples, following by library preparation. Libraries were sequenced on an Illumina MiSeq™ System by Novogene Biotech Co., Ltd. (Beijing, China). Metagenome analysis was performed as previously described (Wu et al., 2024), where *α*- and *β*-diversity indices were calculated at the species levels, and significantly divergent microbial taxa (Differentially abundant microbes in metagenomic, DMMs) were identified using an LDA score threshold >3. Significance thresholds for pathways were set at *p <* 0.05.

### 2.6 Untargeted metabolomics

Fecal and serum sample preparations, mass spectrometry analyses, and data analysis were performed as described in a previous study (Jiang et al., 2024). Differential metabolites (DMs) were identified based on the following thresholds: variable importance in projection (VIP) scores >1, fold change (FC) > 2 or < 0.5, and *p <* 0.05. Significantly enriched pathways were annotated using the KEGG database.

### 2.7 Multiomic (microbiome and metabolome) analysis

The relative abundances of DAMs and DMMs and the relative concentrations of DMs in serum and feces were used to calculate microbe-metabolite relationships through Spearman’s correlation coefficient (R) and a 5% false discovery rate (FDR). Significant pairwise interactions were defined as adjusted *p <* 0.05. Co-occurrence networks were generated using Cytoscape v. 3.8.2 software.^2^

## 3 Results

### 3.1 Physiological and biochemical indices of the Guizhou mini-pig DM model

**Figure 1:**
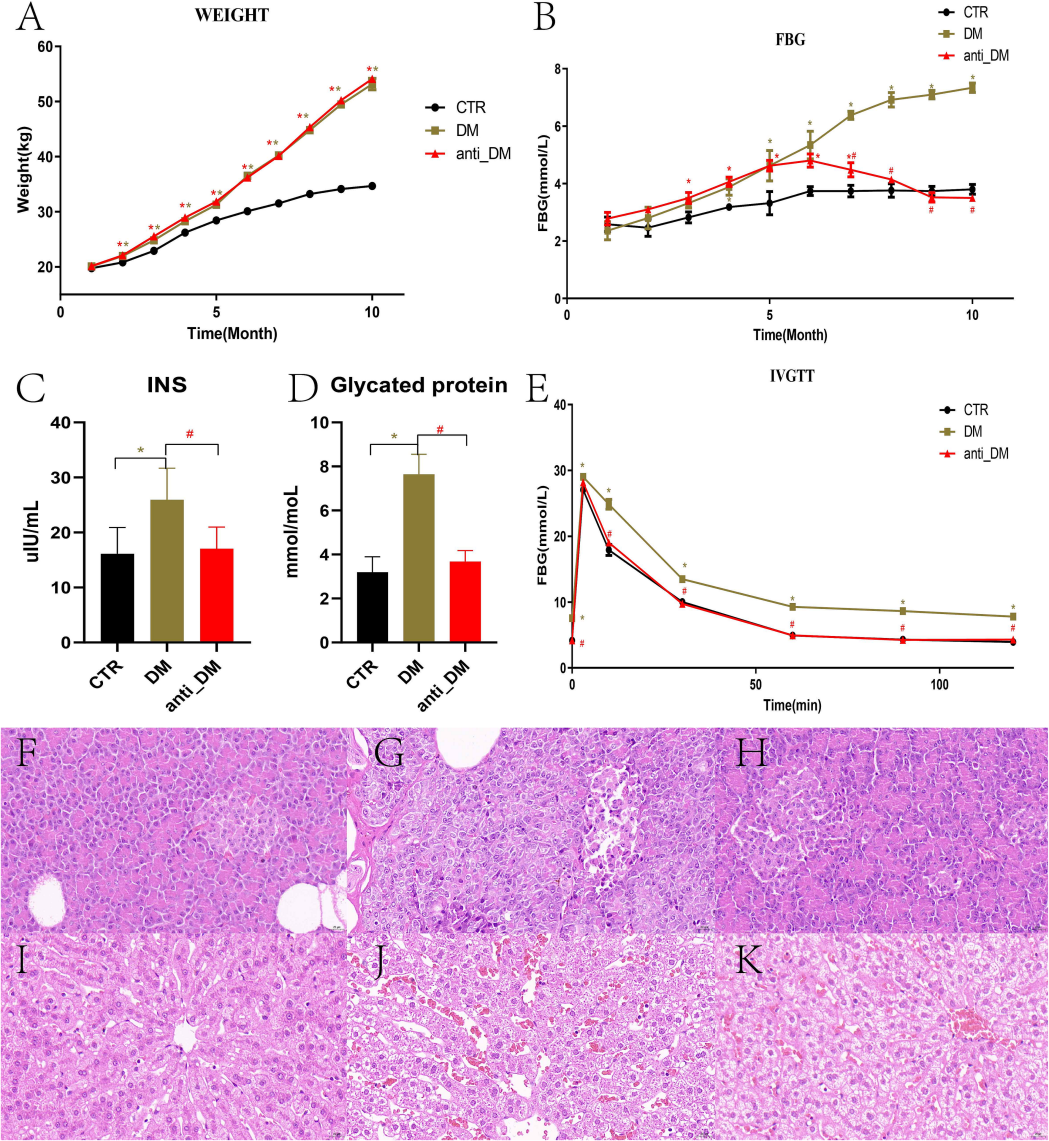
Indices and HE-stained sections from the diabetic model. **(A)** BW; **(B)** FBG concentration; **(C)** INS concentration; **(D)** HbA1C concentration; **(E)** Change to FBG concentrations as determined with the IVGTT; **(F)** HE-stained pancreas sections of the CTR group; **(G)** HE-stained pancreas sections of the DM group; **(H)** HE-stained pancreas sections of the anti-DM group; **(I)** HE-stained liver sections of the CTR group; **(J)** HE-stained liver section of the DM group; **(K)** HE-stained liver section of the anti-DM group.

Among the pigs in the HFD group, five were assigned to the DM group and five to the anti-DM group. The BW of Guizhou mini-pigs in all three groups increased throughout the experiment but remained significantly lower in the CTR group compared to the DM and anti-DM groups from month 2 to 10 (*p <* 0.05) (Figure 1A). No significant BW difference was observed between the DM and anti-DM groups during the experimental period (*p >* 0.05) (Figure 1A). HFD significantly elevated FBG levels in the DM group relative to the CTR group from month 4 to 10 (*p <* 0.05) (Figure 1B). In contrast, the anti-DM group exhibited transient FBG elevation (significantly higher than CTR from month 3 to 7; *p <* 0.05), followed by a progressive decline to CTR-equivalent levels by months 8–10 (p > 0.05 vs. CTR; *p* < 0.05 vs. DM; Figure 1B). Peak FBG levels in the anti-DM group occurred at month 6 (Figure 1B). At month 10, both INS and HbA1c concentrations were significantly elevated in the DM group compared to CTR and anti-DM groups (*p <* 0.05), while no CTR vs. anti-DM difference (*p >* 0.05) (Figures 1C,D). IVGTT revealed sustained hyperglycemia in the DM group (FBG significantly higher than CTR and anti-DM from 10 to 120 min; *p <* 0.05), while CTR and anti-DM groups maintained comparable glucose tolerance (*p >* 0.05) (Figure 1E).

### 3.2 Tissue lesions of the Guizhou mini-pig T2DM model

HE-stained pancreatic sections from the CTR group exhibited intact islet architecture with clearly defined cellular boundaries and minimal stromal adipocyte infiltration (Figure 1F). In contrast, DM group pancreata displayed had structural disorganization, marked islet apoptosis, and diffuse stromal adipocyte deposition (Figure 1G). The anti-DM group demonstrated pancreatic structure and cell arrangements were relatively clear, with some adipocyte aggregation (Figure 1H). Hepatic histology in CTR animals revealed normal hepatocyte cord architecture and absence of lipid vacuolization (Figure 1I). DM group livers showed severe microvesicular steatosis, ballooning degeneration, and loss of zonal organization (Figure 1J). Anti-DM group livers exhibited moderate hepatocyte swelling with patchy macrovesicular steatosis and partial retention of lobular architecture (Figure 1K).

### 3.3 Results of 16 s rRNA sequencing

**Figure 2:**
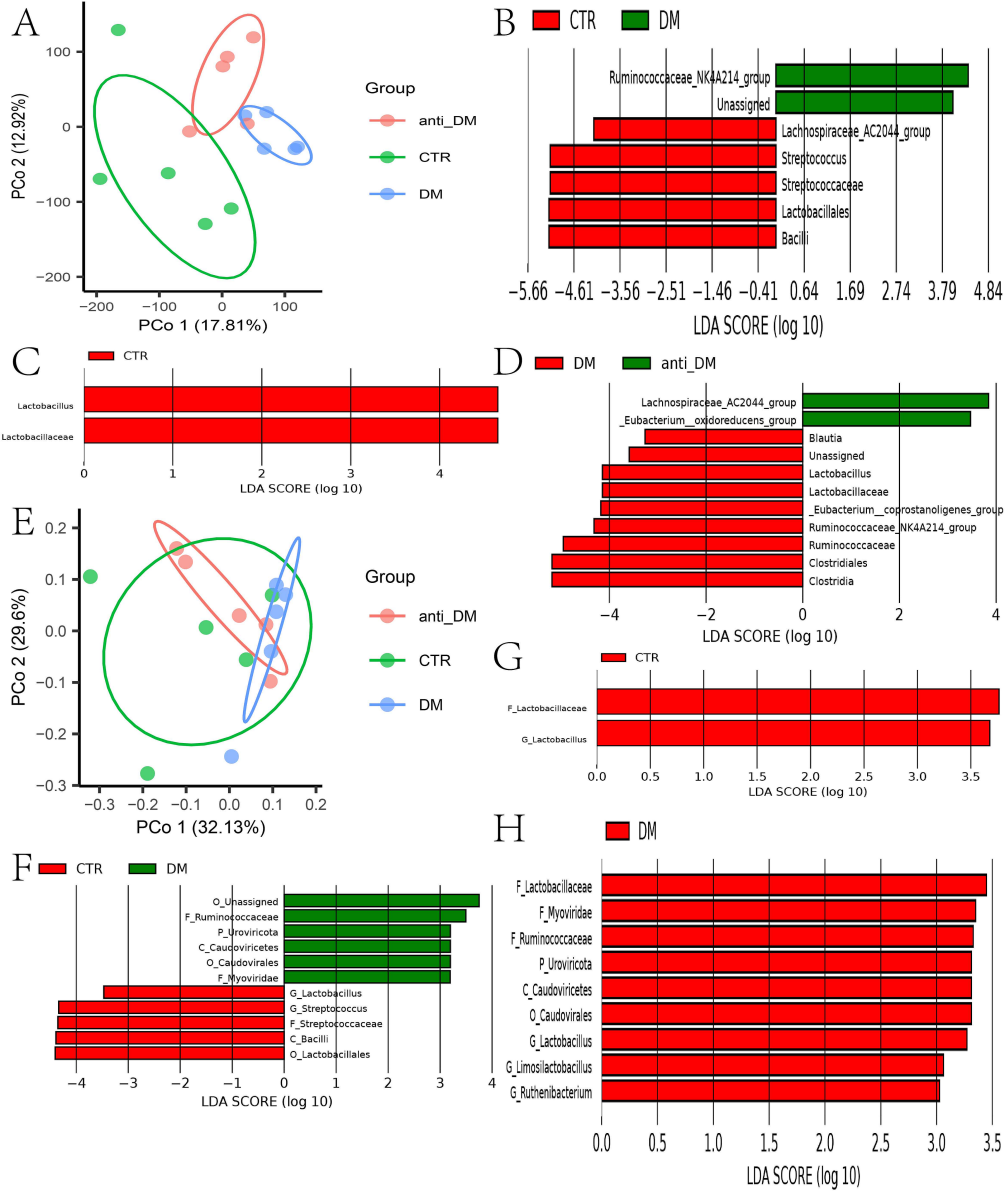
Microbiome analysis. **(A)** Results of *β*-diversity analysis based on 16S rRNA sequences; **(B)** microbes that differed between the CTR and DM groups; **(C)** microbes that differed between the CTR and anti-DM groups; **(D)** microbes that differed between the DM and anti-DM groups. **(E)** β-diversity of the three groups at the species level; **(F)** microbes that differed between the CTR and DM groups at the phylum level; **(G)** microbes that differed between the CTR and DM groups at the genus level; **(H)** microbes that differed between the CTR and anti-DM groups at the genus level; **(I)** microbes that differed between the DM and anti-DM group at the genus level.

No significant differences were observed in fecal microbial *α*-diversity indices (ACE, Chao 1, Simpson, and Shannon) among the three groups (*p* > 0.05) (Supplementary Figures S1A–D). *β*-Diversity analysis revealed distinct clustering of the DM group separate from the CTR group, with the anti-DM group occupying an intermediate position (*p* < 0.001) (Figure 2A). Phylum-level composition analysis demonstrated dominance of Firmicutes (80.68%), Bacteroidetes (10.36%), and Proteobacteria (6.20%) in CTR; Firmicutes (79.86%), Proteobacteria (10.00%), and Bacteroidetes (7.31%) in DM; and Firmicutes (73.92%), Bacteroidetes (10.56%), Proteobacteria (8.91%), and Spirochaetae (5.05%) in anti_DM (Supplementary Figure S1E). At the genus level, the main bacteria were *Streptococcus* (29.37%), *Christensenellaceae R-7 group* (11.33%), *Ruminococcaceae UCG-002* (7.25%), and *Escherichia-Shigella* (5.77%) in CTR; *Christensenellaceae R-7 group* (26.32%), *Ruminococcaceae UCG-002* (11.51%), *Escherichia-Shigella* (9.44%), and *Ruminococcaceae NK4A214 group* (4.88%) in DM; and *Streptococcus* (24.33%), *Christensenellaceae R-7 group* (18.34%), *Escherichia-Shigella* (8.41%), and *Ruminococcaceae UCG-002* (4.93%) in anti-DM (Supplementary Figure S1F). At the genus level, the results of LEfSe analysis showed that the abundances of *Streptococcus* and *Lachnospiraceae AC2044 group* were significantly higher in the CTR group than the DM group (*p <* 0.05), while the abundances of *Ruminococcaceae NK4A214 group* and *unrnak_f Ruminococcaceae* were significantly lower (*p <* 0.05) (Figure 2B). The abundance of *Lactobacillus* was significantly higher in the CTR group than the anti-DM group (*p <* 0.05) (Figure 2C). The abundances of *Lactobacillus*, *Ruminococcaceae NK4A214 group*, *Blautia*, *unrnak_f Ruminococcaceae*, and *Eubacterium coprostanoligenes group* were significantly higher in the DM group than the anti-DM group (*p <* 0.05), while the abundances of *Eubacterium oxidoreducens group* and *Lachnospiraceae AC2044 group* were significantly lower (*p <* 0.05) (Figure 2D).

Secondary KEGG pathway analysis showed that the “Metabolism of Cofactors and Vitamins” and “Cardiovascular Diseases” pathways were significantly higher and lower, respectively, in the DM group than the CTR group (*p <* 0.05) (Supplementary Figure S1G). The “Cardiovascular Diseases” pathway was significantly lower in the anti-DM group than the CTR group (*p <* 0.05) (Supplementary Figure S1H). The “Energy Metabolism” and “Environmental Adaptation” pathways were significantly higher in the DM group than the anti-DM group (*p <* 0.05) (Supplementary Figure S1I). Three-level KEGG pathway analysis showed that eight pathways were significantly lower in the DM group than the CTR group, which included the “Amino sugar and nucleotide sugar metabolism” and “Starch and sucrose metabolism” pathways, while 11 pathways were significantly higher in the DM group than the CTR group (*p <* 0.05) (Supplementary Figure S2A). Eight pathways were significantly lower in the anti-DM group than the CTR group, which included “p53 signaling pathway,” while the “Biosynthesis of type II polyketide backbone” pathway was significantly higher in the anti-DM group than the CTR group (*p <* 0.05) (Supplementary Figure S2B). In addition, 16 pathways were significantly higher in the DM group than the anti-DM group, which included the “mTOR signaling pathway” and “PPAR signaling pathway,” and nine pathways were significantly lower in the DM group than the anti-DM group, such as “Arachidonic acid metabolism” and “Carbohydrate digestion and absorption” (*p <* 0.05) (Supplementary Figure S2C). Notably, the “Cardiovascular Diseases,” “Viral myocarditis,” “Colorectal cancer,” “Toxoplasmosis,” “Influenza A,” “p53 signaling pathway,” and “Small cell lung cancer” pathways were significantly higher in the CTR group than the DM and anti-DM groups (*p <* 0.05).

### 3.4 Metagenomic results

**Figure 3:**
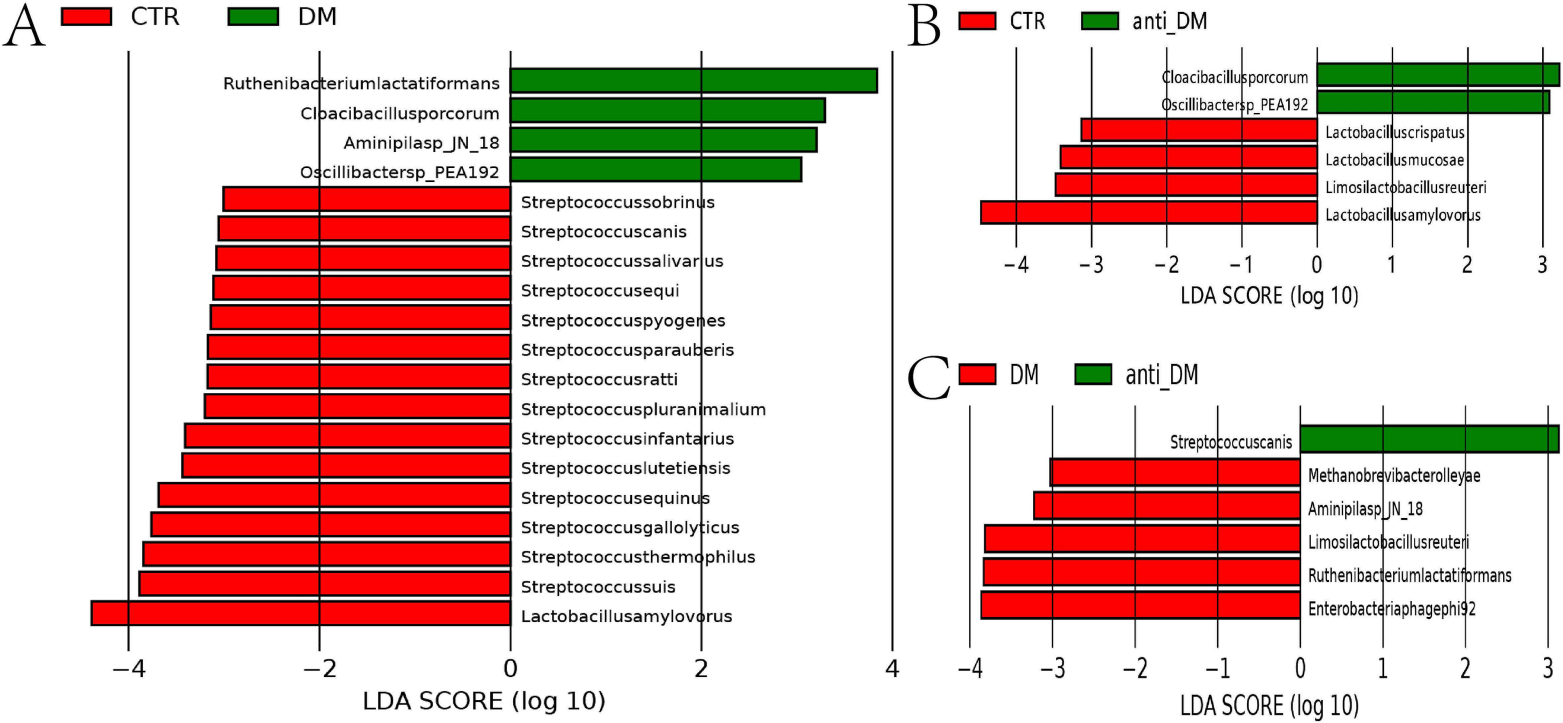
Different microbes among the groups at the genus level. **(A)** microbes that differed between the CTR and DM groups at the species level; **(B)** microbes that differed between the CTR and anti-DM groups at the species level; **(C)** microbes that differed between the DM and anti-DM group at the species level.

Metagenomic sequencing analysis showed that there were no significant *α*-diversity diffferences (ACE, Chao 1, Simpson, and Shannon index) among groups (Supplementary Figures S3A–D), with the anti-DM group positioned intermediately between DM and CTR clusters (Figure 2E). Species-level profiling demonstrated dominant taxa as *Bacteroides fragilis* (9.63%), *Lactobacillus amylovorus* (4.01%), and *Escherichia coli* (1.94%) in CTR; *B. fragilis* (3.77%), *E. coli* (2.94%), and *Phocaeicola vulgatus* (2.38%) in DM; and *B. fragilis* (3.50%), *E. coli* (2.98%), and *Oscillibacter* sp. *NSJ-62* (1.44%) in anti-DM (Supplementary Figure S3E). LEfSe analysis at genus level showed that the abundances of *Streptococcus* and *Lactobacillus* were significantly higher in the CTR group than the DM group (*p <* 0.05) (Figure 2F). At the species level, the results of LEfSe analysis showed that the abundances of 15 microbes were significantly higher in the CTR group than the DM group, which included *L. amylovorus*, *Streptococcus thermophilus*, *Streptococcus suis*, *Streptococcus gallolyticus*, *Streptococcus equinus*, *Streptococcus infantarius*, *Streptococcus lutetiensis*, *Streptococcus pluranimalium*, *Streptococcus ratti*, *Streptococcus parauberis*, *Streptococcus pyogenes*, *Streptococcus equi*, *Streptococcus salivarius*, *Streptococcus canis*, and *Streptococcus sobrinus* (*p <* 0.05) (Figure 3A). The abundances of four microbes were significantly lower in the CTR group than the DM group, which included *Ruthenibacterium lactatiformans*, *Oscillibacter* sp.*PEA192*, *Aminipila* sp.*JN18*, and *Cloacibacillus porcorum* (*p <* 0.05) (Figure 3A). At the genus level, the abundance of *Lactobacillus* was significantly higher in the CTR group than the anti-DM group (*p <* 0.05) (Figure 2G). At the species level, the abundances of four microbes were significantly higher in the CTR group than the anti-DM group, which included *L. amylovorus*, *Limosilactobacillus reuteri*, *Lactobacillus mucosae*, and *Lactobacillus crispatus* (*p <* 0.05) (Figure 3B), while the abundances of *Oscillibacter* sp.*PEA192* and *Cloacibacillus porcorum* were significantly lower (*p <* 0.05) (Figure 3B). At the genus level, the abundances of *Lactobacillus*, *Ruthenibacterium*, and *Limosilactobacillus* were significantly higher in the DM group than the anti-DM group (*p <* 0.05) (Figure 2H). At the species level, the abundances of five microbes were significantly higher in the DM group than the anti-DM group, which included *L. reuteri*, *R. lactatiformans*, *Aminipila* sp.*JN18*, *Enterobacteria phagephi 92*, and *Methanobrevi bacterolleyae* (*p <* 0.05) (Figure 3C), while the abundance of *S. canis* was significantly higher (*p <* 0.05) (Figure 3C).

Secondary KEGG pathway analysis showed that five pathways were significantly higher in the DM group than the CTR group, which included “Aging” and “Cell growth and death” (*p <* 0.05) (Supplementary Figure S3F). The “Protein families: metabolism” pathway was significantly lower in the anti-DM group than the CTR group (*p <* 0.05) (Supplementary Figure S3G). The secondary KEGG pathways were significantly higher in the DM group than the anti-DM group, which included as “Aging” and “Signaling molecules and interaction” (*p <* 0.05) (Supplementary Figure S3H). The results of three-level KEGG pathway analysis showed that 10 pathways were significantly lower in the DM group than the CTR group, which included “Galactose metabolism” and “Histidine metabolism,” and 23 pathways were significantly higher in the DM group than the CTR group, which included “Insulin signaling pathway,” “AMPK signaling pathway,” and “Cell growth” (*p <* 0.05) (Supplementary Figure S4A). Four pathways were significantly lower in the anti-DM group than the CTR group, while two pathways were significantly higher (*p <* 0.05) (Supplementary Figure S4B). In addition, 35 pathways were significantly higher in the DM group than the anti-DM group, which included “Carbohydrate metabolism,” “Fatty acid degradation,” and “Cholesterol metabolism,” and 17 pathways were significantly lower, such as “Fructose and mannose metabolism,” and “Fatty acid biosynthesis” (*p <* 0.05) (Supplementary Figure S5). Notably, the “Bacterial secretion system” pathway was significantly lower in the CTR group than the DM and anti-DM groups (*p <* 0.05).

### 3.5 Candidate microbes associated with HFD, T2DM, and disease resistance

Key differential taxa identified by metagenomic sequencing and 16S rRNA analysis included the *Ruminococcaceae NK4A214 group* and *Streptococcus* (CTR vs. DM), *Lactobacillus* (CTR vs. anti-DM), and *Lactobacillus* with *Ruminococcaceae NK4A214 group* (anti-DM vs. DM). Interestingly, the abundances of *Lactobacillus* and *L. amylovorus* were significantly higher in the CTR group than the DM and anti-DM groups (*p <* 0.05), while the abundance of *Lactobacillus* was significantly higher in the DM group than the anti-DM group (*p <* 0.05). Conversely, the abundances of *Oscillibacter* sp.*PEA192* and *C. porcorum* were significantly lower in the CTR group than the DM and anti-DM groups (*p <* 0.05). These findings nominate *Lactobacillus*, *L. amylovorus*, *Oscillibacter* sp.*PEA192*, and *C. porcorum* as HFD-responsive microbial signatures in the Guizhou mini-pig model. Further analysis revealed that the abundance of *L. reuteri* was significantly lower in the anti-DM group than the CTR and DM groups (*p <* 0.05), suggesting its protective role in T2DM resistance. The abundance of *Ruminococcaceae NK4A214 group* was significantly higher in the DM group than the CTR and anti-DM groups (*p <* 0.05), while the abundance of *S. canis* was significantly lower. The abundances of *R. lactatiformans* and *Aminipila* sp.*JN18* were significantly higher in the DM group than the CTR and anti-DM groups (*p <* 0.05). These findings implicated that the *Ruminococcaceae NK4A214 group*, *S. canis*, *R. lactatiformans*, and *Aminipila* sp.*JN18* are take part in T2DM pathogenesis.

### 3.6 Candidate metabolites of HFD, T2DM, and T2DM resistance

**Figure 4:**
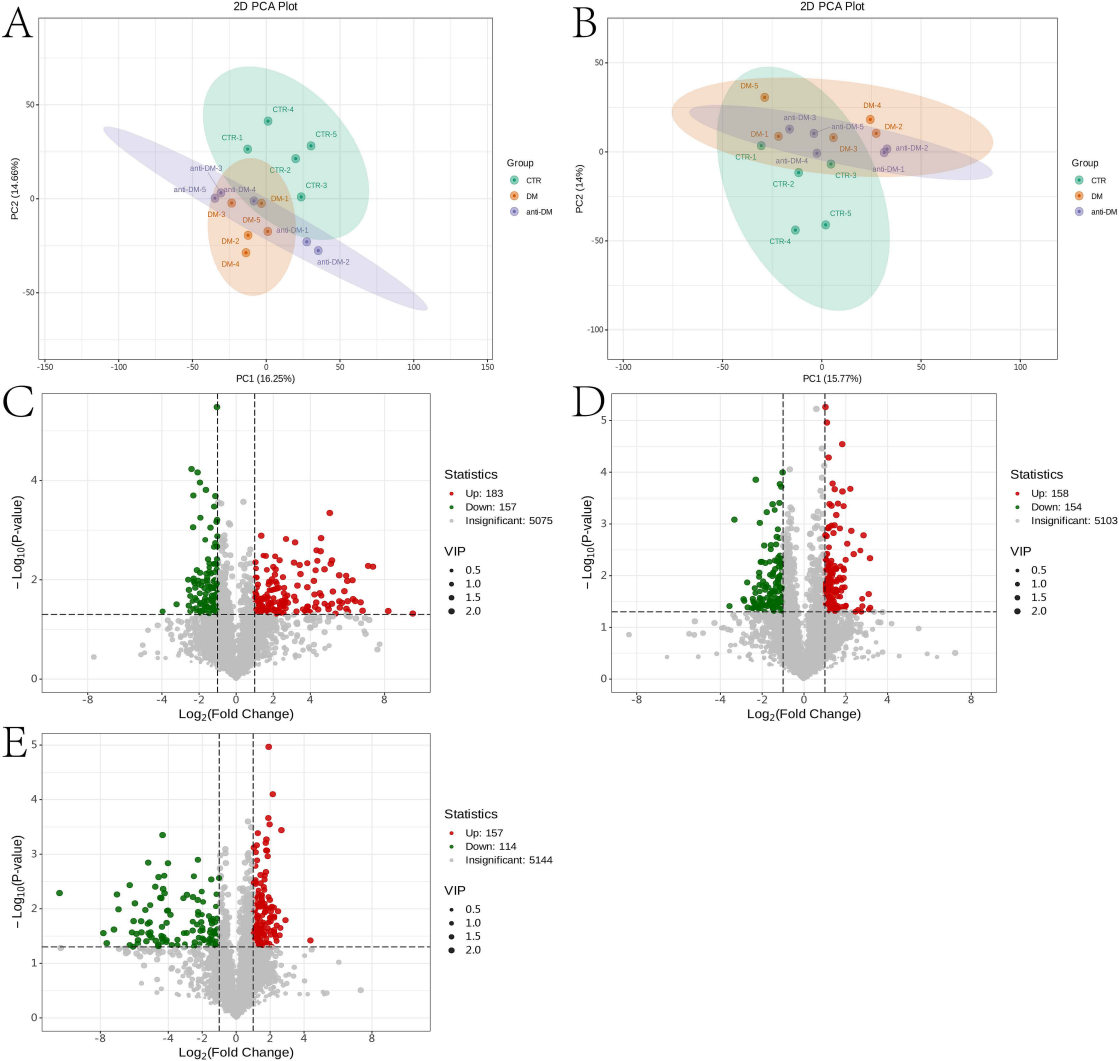
Metabolome analysis of fecal samples. **(A)** PCA plot of the positive ion mode of the three groups; **(B)** PCA plot of the negative ion mode of the three groups; **(C)** differential metabolites between the DM and CTR groups; **(D)** differential metabolites between the anti-DM and CTR groups; **(E)** differential metabolites between the anti-DM and DM groups.

**Figure 5:**
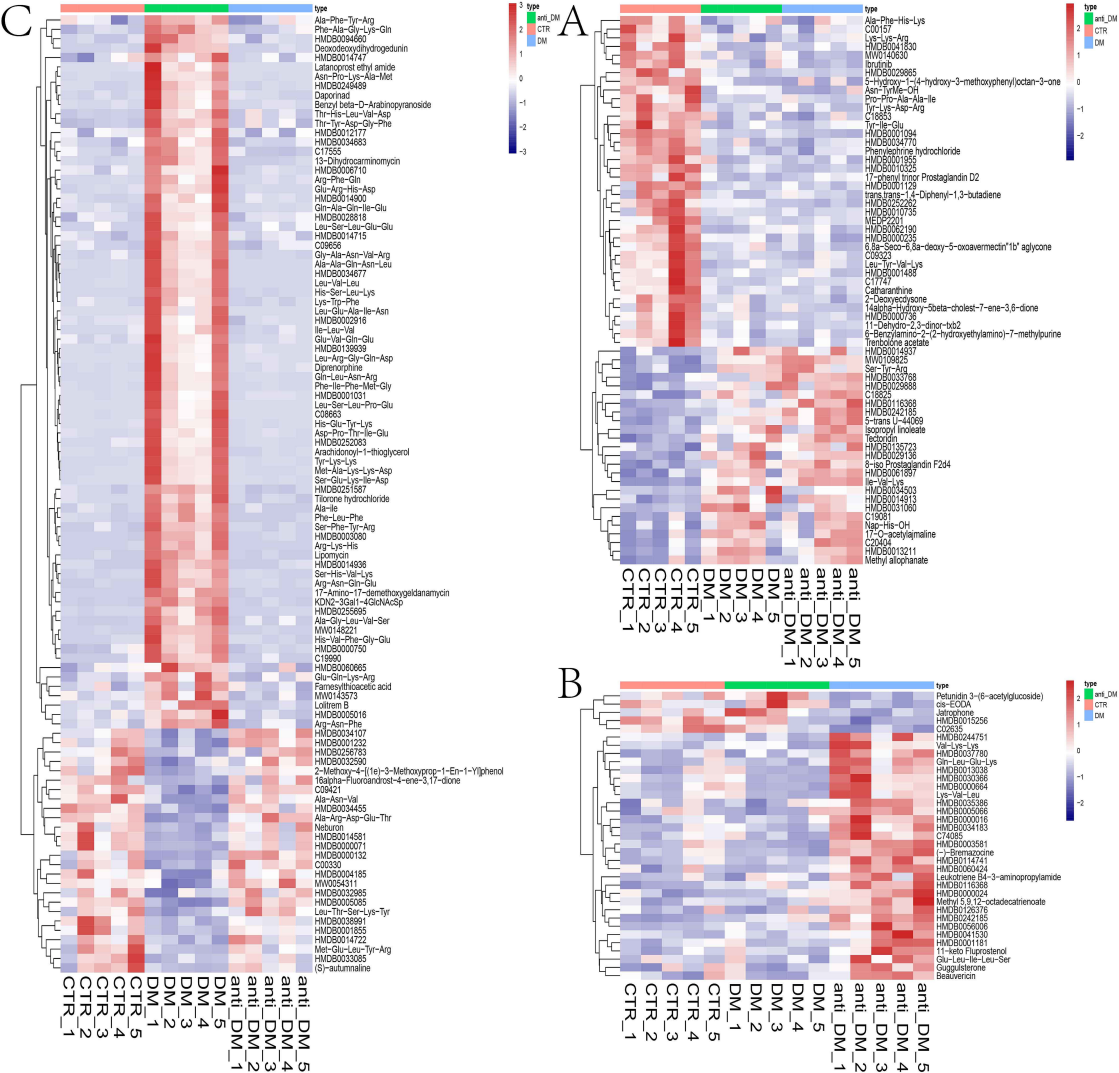
Candidate metabolites in the Guizhou mini-pig. **(A)** Candidate metabolites associated with HFD; **(B)** candidate metabolites associated with T2DM resistance; **(C)** candidate metabolites associated with T2DM.

**Figure 6:**
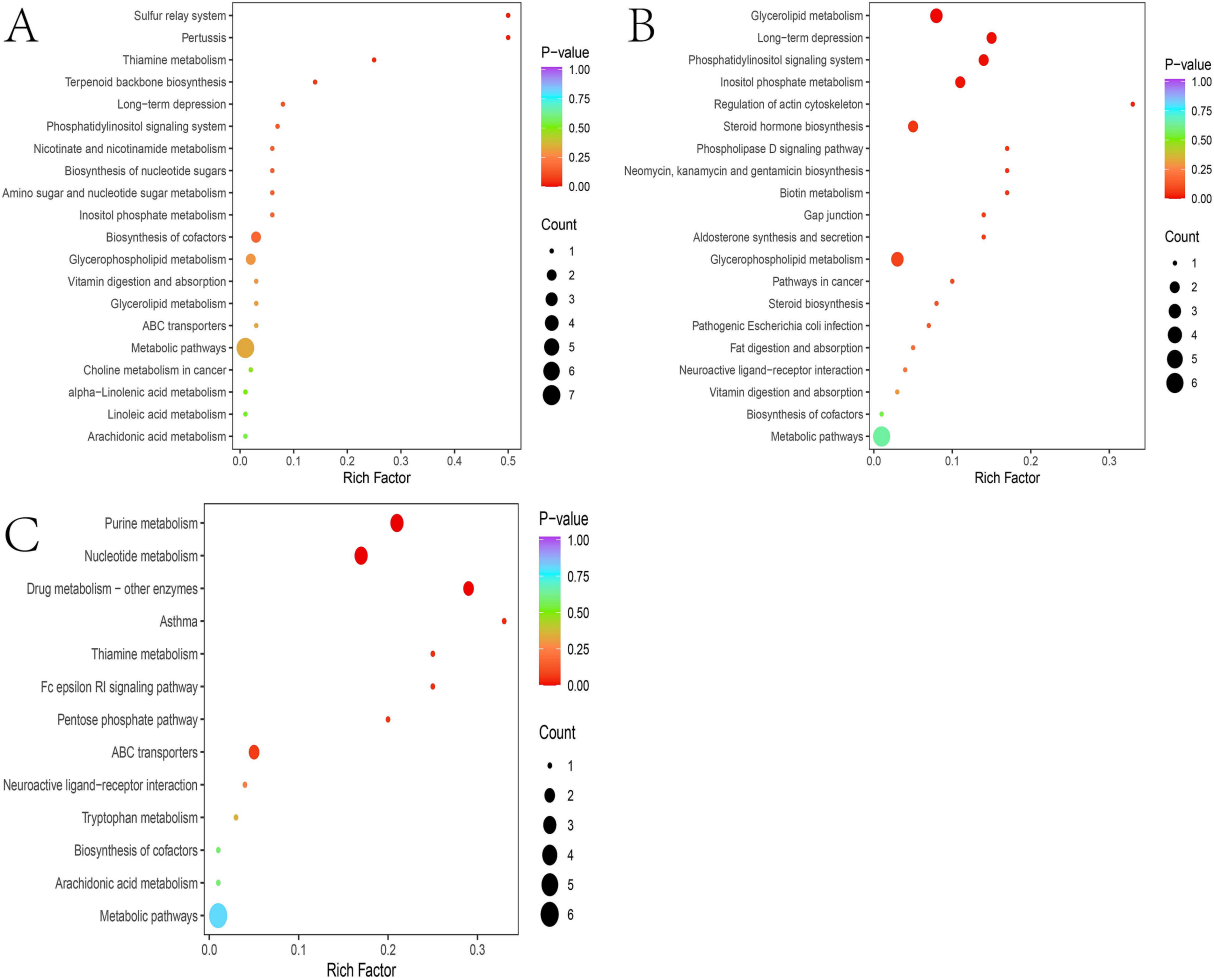
Pathways associated with the candidate metabolites in the Guizhou mini-pig. **(A)** Pathways associated with candidate metabolites related to HFD; **(B)** pathways associated with candidate metabolites related to T2DM resistance; **(C)** pathway associated with candidate metabolites related to T2DM.

Metabolomics analysis showed clear separation of the CTR group from DM and anti_DM groups via PCA (Figures 4A,B). In total, levels of 157 metabolites were significantly lower in the CTR group than the DM group (*p <* 0.05) (Figure 4C and Supplementary Table S1) and significantly enriched in the “Drug metabolism - other enzymes” and “Caffeine metabolism” pathways (*p <* 0.05) (Supplementary Figure S6A), while levels of 183 metabolites were significantly higher in the CTR group than the DM group (*p <* 0.05) (Figure 4C and Supplementary Table S1) and significantly enriched in six KEGG pathways, including “Thiamine metabolism,” “Purine metabolism,” “Nucleotide metabolism,” “Renal cell carcinoma,” “Pertussis,” and “Sulfur relay system” (*p <* 0.05) (Supplementary Figure S6B). In total, the levels of 158 metabolites were significantly lower in the CTR group than the anti-DM group (*p <* 0.05) (Figure 4D and Supplementary Table S1), and significantly enriched in 38 KEGG pathways, such as “Regulation of lipolysis in adipocytes,” “Glycerolipid metabolism,” “Fat digestion and absorption,” “Insulin secretion,” and “Insulin signaling pathway” (*p <* 0.05) (Supplementary Figure S6C). Also, the levels of 154 metabolites were significantly higher in the CTR group than the DM group (*p <* 0.05) (Figure 4D and Supplementary Table S1) and significantly enriched in the “Antifolate resistance” pathway (*p <* 0.05) (Supplementary Figure S6D). Meanwhile, levels of 157 metabolites were significantly lower in the DM group than the anti-DM group (*p <* 0.05) (Figure 4E and Supplementary Table S1) and significantly enriched in the “Nucleotide metabolism,” “Purine metabolism,” and “Glycerolipid metabolism” pathways (*p <* 0.05) (Supplementary Figure S6E), and levels of 114 metabolites were significantly higher in the DM group than the anti-DM group (*p <* 0.05) (Figure 4E and Supplementary Table S1) and significantly enriched in five KEGG pathways, which included the “Drug metabolism - other enzymes,” “Pantothenate and CoA biosynthesis,” “Phototransduction,” “Retinol metabolism,” and “Asthma” pathways (*p <* 0.05) (Supplementary Figure S6F). Interestingly, levels of 25 metabolites were significantly lower in the CTR group than the DM and anti-DM groups, while levels of 38 metabolites were significantly higher (*p <* 0.05) (Figure 5A). In total, 63 metabolites were associated with HFD-induced T2DM in the Guizhou mini-pig and participated in 21 KEGG pathways, such as “Glycerophospholipid metabolism,” “Glycerophospholipid metabolism,” “Alpha-Linolenic acid metabolism,” “Linoleic acid metabolism,” and “Arachidonic acid metabolism” (Figure 6A). Meanwhile, levels of 30 metabolites were significantly higher in the anti-DM group than the CTR and DM groups, while levels of five metabolites were significantly lower (*p <* 0.05) (Figure 5B). In total, 35 metabolites were associated with T2DM resistance in the Guizhou mini-pig and participated in 20 KEGG pathways, such as “Glycerolipid metabolism,” “Glycerophospholipid metabolism,” “Fat digestion and absorption,” and “Vitamin digestion and absorption” (Figure 6B). Besides, levels of 76 metabolites were significantly higher in the DM group than the CTR and anti-DM groups, while levels of 26 metabolites were significantly lower (*p <* 0.05) (Figure 5C). In total, 102 metabolites were associated with T2DM in the Guizhou mini-pig and participated in 13 KEGG pathways, such as “Purine metabolism,” “Thiamine metabolism,” “Pentose phosphate pathway,” and “Arachidonic acid metabolism” (Figure 6C).

### 3.7 Co-occurrence network of fecal microbiota and metabolites

**Figure 7:**
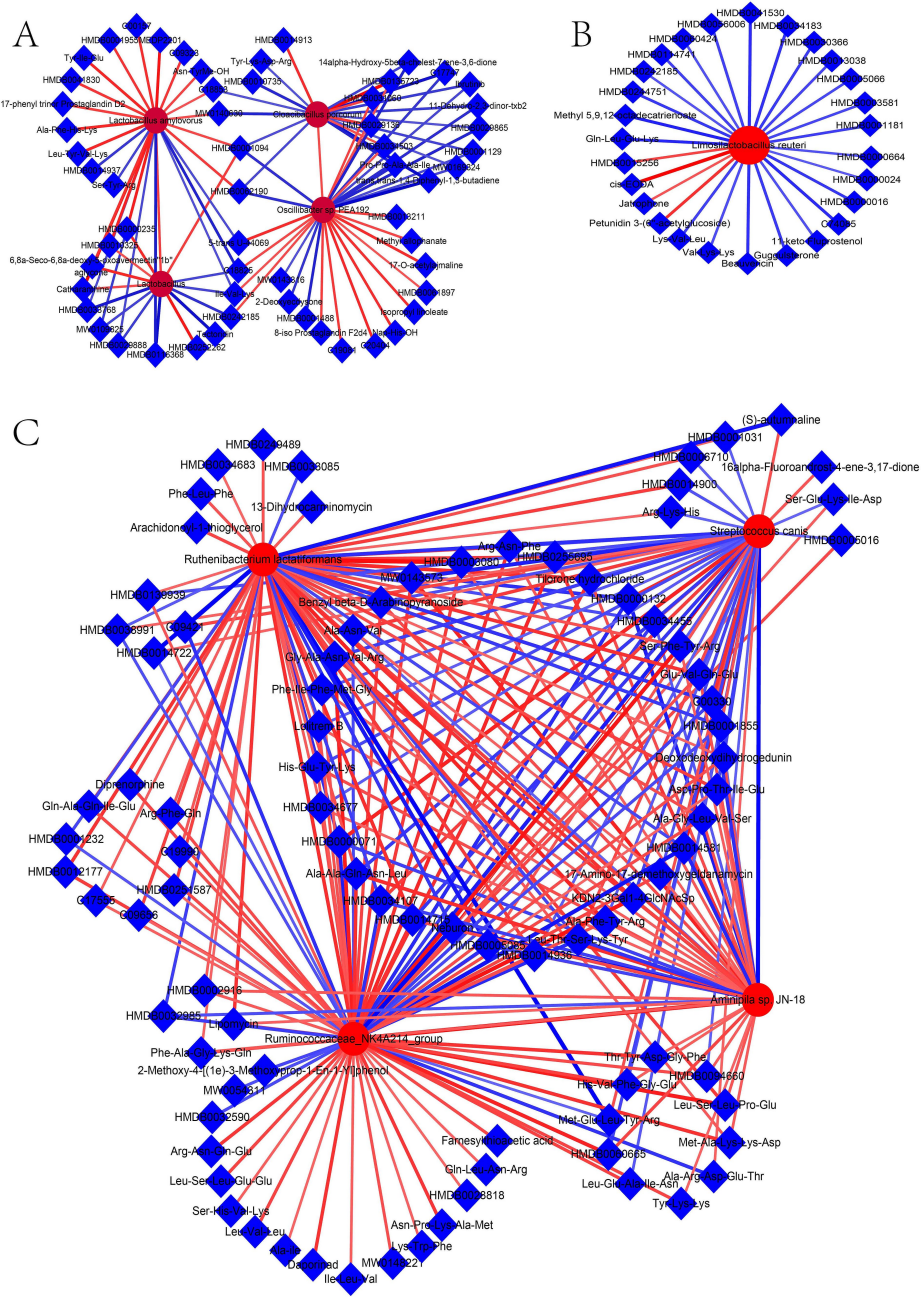
Networks of HFD, T2DM resistance, and T2DM. **(A)** HFD network; **(B)** T2DM network; **(C)** T2DM resistance network.

Analysis of the microbiome identified four, one, and four microbes associated with HFDs, T2DM resistance, and T2DM, respectively, in the Guizhou mini-pig. Meanwhile, 63, 35, and 102 metabolites were found to correlate with HFDs, T2DM resistance, and T2DM, respectively. Spearman correlation analysis between candidate microbes and metabolites associated with HFDs revealed 92 significant microbe-metabolite pairs, with correlation coefficients ranging from −0.58 to −0.84 and 0.58 to 0.86 (Figure 7A). Specifically, *Lactobacillus*, *L. amylovorus*, *Oscillibacter* sp.*PEA192*, and *C. porcorum* were associated with 14, 27, 31, and 20 metabolites, respectively. For T2DM-associated candidate microbes and metabolites, Spearman analysis identified 238 microbe-metabolite pairs with coefficients ranging from −0.54 to −0.90 and 0.54 to 0.88 (Figure 7B). While *Lactobacillus*, *L. amylovorus*, *Oscillibacter* sp.*PEA192*, and *C. porcorum* maintained associated with 14, 27, 31, and 20 metabolites, respectively. *Ruminococcaceae NK4A214 group*, *S. canis*, *R. lactatiformans*, and *Aminipila* sp.*JN18* demonstrated associations with 78, 52, 64, and 44 metabolites, respectively. Finally, Spearman analysis of candidate microbes and *L. reuteri* in relation to T2DM resistance identified 27 microbe-metabolite pairs, with correlation coefficients spanning −0.56 to −0.75 and 0.58–0.88 (Figure 7C).

## 4 Discussion

T2DM Models have been established using miniature pig breeds including Bama (Yan et al., 2018), Ossabaw (Badin et al., 2018), Yucatan (Johnson et al., 2021), and Wuzhishan (Niu et al., 2020). Our group previously developed a Guizhou mini-pig T2DM model through streptozocin injection (Wu et al., 2024). However, while STZ injection and high-fat diets demonstrated incomplete consistency in their effects (Wu et al., 2018), HFD implementation more closely mimics human T2DM pathophysiology. Consequently, this study established a Guizhou mini-pig T2DM model using the HFD approach. The HFD regimen progressively increased BW and FBG from month 3 to 10, aligning with previous study (Wu et al., 2018). Significant differences emerged in FBG, INS, HbA1C and IVGTT indices between DM and CTR groups, confirming successful model establishment (Chen et al., 2009), albeit with a success rate of only 16.67% (5/30). This rate substantially trailed the Bama miniature pig’s 66.67% (10/15) success rate while approximating the Duroc pig’s 13.33% (2/15) (Zhang L. et al., 2019), differences potentially attributable to long-term inbreeding and feeding protocols. Notably, anti-DM group FBG levels declined from month 6 onward, with month 10 measurements of FBG, INS, HbA1c, and IVGTT indices approaching normal ranges. Structural and morphological analyses further revealed pancreatic and hepatic tissue damage in DM group pigs, consistent with prior observations (Zhao et al., 2023). Intriguingly, anti-DM group pigs exhibited relatively mild tissue damage but developed fatty liver pathology. These findings underscore the need for further research to identify biomarkers of both T2DM progression and resistance mechanisms in the Guizhou mini-pig model.

Metagenomic sequencing combined with 16S rRNA analysis of CTR, DM, and anti-DM groups showed that while the HFD exerted limited effects on *α*-diversity indices, it significantly altered microbiome structure and composition, aligning with previous findings (Niu et al., 2022). Notably, *β*-diversity indices showed similarity between anti-DM and CTR groups, consistent with their comparable physiological parameters, biochemical profiles, and tissue morphology. Furthermore, microbes and pathways differences related to T2DM were pronounced between CTR and DM groups than between CTR and anti-DM groups, mirroring phenotypic variations. Importantly, *Lactobacillus*, *L. amylovorus*, *Oscillibacter* sp.*PEA192*, and *C. porcorum* were associated with the HFD used in this study. While Wang et al. (2020) observed HFD-induced reduction of *Lactobacillus* abundance in mice - consistent with our results—Park et al. (2019) demonstrated *L. amylovorus KU4* anti-obesity effects in HFD models. Contrastingly, Mobini et al. (2017) reported *L. reuteri DSM 17938* improved INS sensitivity in T2DM patients, while Abot et al. (2024) found *L. reuteri BIO7251* reduced glucose absorption in diabetic mice. Hsieh et al. (2018) Documented beneficial effect of *L. reuteri strains ADR-1*/ *ADR-3* in T2DM patients and *GL-104*‘s ability to lower FBG and enhance glucose tolerance in murine models (Hsieh et al., 2020). Simon et al. (2015) Further demonstrated *L. reuteri*‘s INS-secretory enhancement in humans. As beneficial gut microbiota, *Lactobacillus* species combat obesity and T2DM through anti-inflammatory mechanisms, endothelial function improvement, and microbiome balance restoration (Song et al., 2021; Kang et al., 2022). Paradoxically, *L. reuteri* abundance in the anti_T2DM group was lower than in both DM and CTR groups, contradicting established literature, necessitating further investigation into its role in T2DM resistance. These findings collectively suggest HFD-induced obesity mechanisms involve reduced *Lactobacillus* and *L. amylovorus* abundance, while diminished *L. reuteri* levels may contribute to T2DM resistance in Guizhou mini-pigs. Additionally, *Ruminococcaceae NK4A214 group*, *S. canis*, *R. lactatiformans*, and *Aminipila* sp.*JN18* demonstrated T2DM associations. A large-scale study identified negative correlations between *Ruminococcaceae NK4A214 group* and INS resistance (Chen Z. et al., 2021), corroborated by Mexican cohort data (Esquivel-Hernández et al., 2023), but conflicting with murine research showing positive correlations (Zhang et al., 2018). Furthermore, the *Ruminococcaceae NK4A214 group* has been identified as a short-chain fatty acid (SCFA) producer, with deficient SCFA production being associated with T2DM pathogenesis (Zhao et al., 2018). The roles of *L. reuteri* in T2DM resistance requires further investigation.

The results of PCA were similar between the anti-DM and CTR groups, consistent with the microbiome analysis results. The number of different metabolites was greater between the CTR and DM groups (340) than between the CTR and anti-DM groups (312), mirroring microbiome findings. “Insulin secretion” and “Insulin signaling pathway” were significantly enriched in the anti-DM group compared to the CTR group. Importantly, 63, 35, and 102 metabolites were associated with HFD, T2DM resistance, and T2DM, respectively, in the Guizhou mini-pig. Moreover, 14 candidate HFD-associated metabolites, including fingolimod, polyoxyethylene sorbitan monooleate, and thiamine, showed significant correlations with *Lactobacillus*. Fingolimod was reported to inhibit HFD-induced weight gain and improve INS sensitivity in mice through changing macrophage-related chronic inflammation gene expression (e.g., *F4/80*, *CD68*, *IL-6*, *TNF-α*, and *MCP-1*) in adipose and hepatic tissues (Yan et al., 2016). Polyoxyethylene sorbitan monooleate, frequently used as a medium supplement for *Lactobacillus* growth (Reitermayer et al., 2018), can induced obesity via altering gut microbial composition/function and increasing pro-inflammatory potential (Chassaing et al., 2015). Thiamine decreased the BW of HFD mice by enhancing metabolism-related enzyme activities (Zheng et al., 2018) and improved *Lactobacillus acidophilus LA-5* growth (Qiao et al., 2024). Meanwhile, 27 HFD-associated metabolites, including atrazine, polyoxyethylene sorbitan monooleate, and thiamine, correlated with *L. amylovorus*. Atrazine showed positive associations with increased T2DM odds (Wei et al., 2023), consistent with our finding. Notably, *Lactobacillus, L. amylovorus*, fingolimod, polyoxyethylene sorbitan monooleate, thiamine, and atrazine constituted key components of the HFD-associated network in Guizhou mini-pigs, indicating these two bacterial taxa and four metabolites as critical nodes in HFD-induced metabolic disorders in this model.

There were 27 candidate HFD metabolites showed significant correlations with *L. reuteri*, including N-Oleoyl-L-Serine, Tolbutamide, Tetradecanoyl carnitine, 3’-Sulfogalactosylceramide, and Guggulsterone. The content of N-oleoyl-L-serine was significantly lower in treatment group rats than in the T2DM group (Ea et al., 2023). As a widely used T2DM medication (Sá et al., 2022), tolbutamide promotes insulin secretion by inhibiting ATP-sensitive potassium channel (Burkart et al., 2024). Metformin-exposed groups exhibited higher tetradecanoyl carnitine levels than controls (Estrella et al., 2021), with this metabolite affecting metabolic health through inflammation and mitochondrial dysfunction (Ho et al., 2024). 3’-Sulfogalactosylceramide demonstrated preventive effects against T2DM in non-obese diabetic mice (Buschard et al., 2001). Guggulsterone exerted hypoglycemic and hypolipidemic effects in T2DM mice via enhancing PPARγ expression and inhibiting preadipocyte differentiation (Sharma et al., 2009). Notably, *L. reuteri*, and teh metabolites N-oleoyl-L-serine, tolbutamide, tetradecanoyl carnitine, 3′-sulfogalactosylceramide, and guggulsterone constituted key components of the T2DM resistance network in Guizhou mini-pigs, identifying this bacterial species and five metabolites as critical nodes in T2DM resistance mechanisms for this model.

Furthermore, 78 T2DM-associated metabolites showed significant correlations with the *Ruminococcaceae NK4A214 group*, including diethyl phthalate, zingerone, enalapril, 5-hydroxytryptophol, 2′-deoxyinosine, icariin, and emetine. Dietary exposure to diethyl phthalate impaired INS signaling in mice, inducing INS resistance by disrupting hepatic and adipose tissue metabolic functions (Mondal and Mukherjee, 2020). Zingerone reduced glucose and INS levels in T2DM mice through enhancing glutathione concentration, antioxidant capacity, and free radical scavenging (Anwer et al., 2021). Enalapril lowered T2DM incidence in patients with impaired FBG by inhibiting angiotensin-converting enzyme, increasing islet blood flow, and improving *β*-cell insulin secretion (Vermes et al., 2003). Pineal gland 5-hydroxytryptophol levels were significantly reduced in T2DM pigs compared to controls (Lewczuk et al., 2018), while testicular 2′-deoxyinosine content was lower in treatment groups versus T2DM groups (Jin et al., 2024). Icariin, a potential T2DM therapeutic, improved pancreatic cell function (Jiang et al., 2023) via activation of the AMPK/GLUT-4 pathway (Li et al., 2020). Emetine emerged as a T2DM treatment candidate by inhibiting NF-κB signaling activation and reducing IL-18/CCL5 expression (Lin et al., 2023). Notably, the *Ruminococcaceae NK4A214 group* and metabolites diethyl phthalate, zingerone, enalapril, 5-hydroxytryptophol, 2′-deoxyinosine, icariin, and emetine formed core components of the Guizhou mini-pig T2DM network, identifying this microbial taxon and seven metabolites as pivotal nodes in T2DM pathogenesis in this model.

Collectively, our study established networks linking HFD, T2DM resistance, and T2DM development in the Guizhou mini-pig, significantly advancing the understanding of T2DM pathogenesis in this HFD-induced model. The candidate microbes and metabolites associated with these networks represent valuable targets for further investigation, notably *L. reuteri* role in T2DM resistance and *Ruminococcaceae NK4A214 group*‘s involvement in T2DM progression.

## 5 Conclusion

This study successfully established a Guizhou mini-pig model of T2DM via HFD intervention. Integrated multi-omics analysis revealed distinct microbe-metabolite networks for HFD, T2DM resistance, and T2DM. *Lactobacillus, L. amylovorus*, *L. reuteri*, and *Ruminococcaceae NK4A214 group* emerged as key microbial components associated with HFD-induced T2DM. Polyoxyethylene sorbitan monooleate, thiamine, atrazine, umbelliferone, nicotinic acid, ibrutinib, N-oleoyl-L-serine, tolbutamide, tetradecanoyl carnitine, 3′-sulfogalactosylceramide, guggulsterone, diethyl phthalate, zingerone, enalapril, 5-hydroxytryptophol, 2′-deoxyinosine, icariin, and emetine were identified as pivotal mediators in this pathogenesis. Collectively, these microbe-metabolite networks in the Guizhou mini-pig provide critical insights into gut microbiota functions underlying T2DM progression.

## Data Availability

The datasets generated and analyzed in the present study are available at the China National Center for Bioinformation: CRA017485.

## Footnotes

^1^ https://diabetesatlas.org/

^2^ https://cytoscape.org/

## Supplementary material

Supplementary Figure S1 The result of 16S rRNA sequencing. **(A)** the ACE index in 16S RNA; **(B)** the Chao 1 index in 16S RNA; **(C)** the Simpson index in 16S RNA; **(D)** the shannon index in 16S RNA; **(E)** the species composition in genus levels; **(F)** the species composition in species levels; **(G)** the secondary KEGG pathway that differed between the CTR and DM groups; **(H)** the secondary KEGG pathway that differed between the CTR and anti_DM groups; **(I)** the secondary KEGG pathway that differed between the DM and anti_DM groups.

Supplementary Figure S2 The difference three-level KEGG pathway of 16S rRNA sequencing. **(A)** The three-level KEGG pathway that differed between the CTR and DM groups; **(B)** the three-level KEGG pathway that differed between the CTR and anti_DM groups; **(C)** the three-level KEGG pathway that differed between the DM and anti_DM groups.

Supplementary Figure S3 The result of metagenome sequencing. **(A)** The ACE index in metagenome; **(B)** the Chao 1 index in metagenome; **(C)** the Simpson index in metagenome; **(D)** the Shannon index in metagenome; **(E)** the species composition in genus levels; F: the secondary KEGG pathway that differed between the CTR and DM groups; **(G)** the secondary KEGG pathway that differed between the CTR and anti_DM groups; **(H)** the secondary KEGG pathway that differed between the DM and anti_DM groups.

Supplementary Figure S4 The difference three-level KEGG pathway of metagenome sequencing. **(A)** The three-level KEGG pathway that differed between the CTR and DM groups; **(B)** the three-level KEGG pathway that differed between the CTR and anti_DM groups.

Supplementary Figure S5 The three-level KEGG pathway that differed between the DM and anti_DM groups.

Supplementary Figure S6 The different metabolites of feces metabolome. **(A)** The significant up-regulated different metabolites between CTR group and DM group; **(B)** the significant down-regulated different metabolites between CTR group and DM group; **(C)** the significant up-regulated different KEGG pathway between CTR group and anti_DM group; **(D)** the significant down-regulated different KEGG pathway between CTR group and anti_DM group; **(E)** the significant up-regulated different metabolites between anti_DM group and DM group; **(F)** the significant down-regulated different metabolites between anti_DM group and DM group.

Supplementary Table S1 The difference metabolites in three groups.
