# Supplementary figures and images for: Microbiome and metabolome explain the high-fat diet-induced diabetes development and diabetes resistance in Guizhou mini-pigs
Source: Front Microbiol. 2025 Apr 9;16:1555069. doi: 10.3389/fmicb.2025.1555069 (PMC12023756; doi:10.3389/fmicb.2025.1555069)

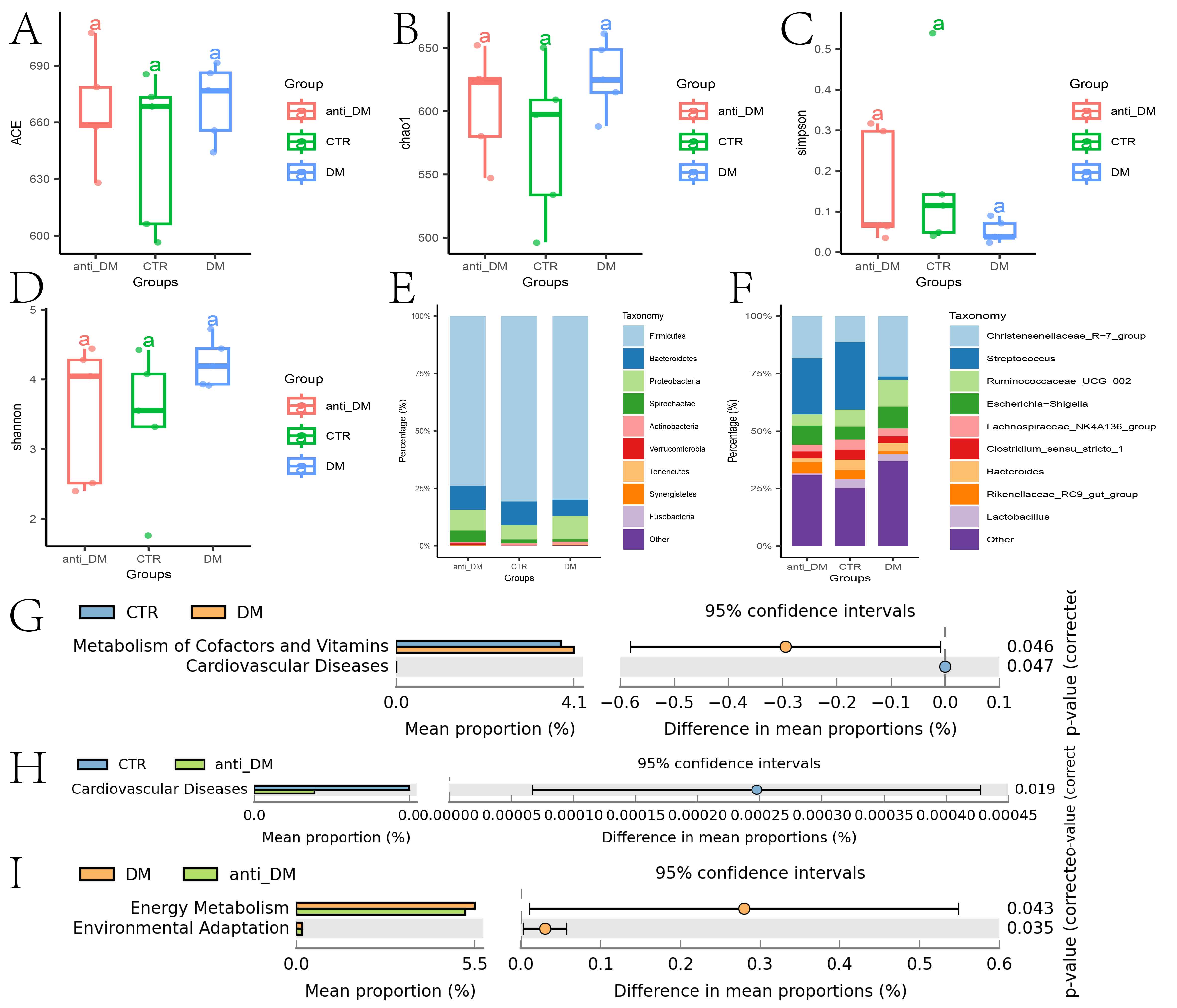

Supplement: Supplementary Figure S1 — The result of 16S rRNA sequencing. (A) the ACE index in 16S RNA; (B) the Chao 1 index in 16S RNA; (C) the Simpson index in 16S RNA; (D) the shannon index in 16S RNA; (E) the species composition in genus levels; (F) the species composition in species levels; (G) the secondary KEGG pathway that differed between the CTR and DM groups; (H) the secondary KEGG pathway that differed between the CTR and anti_DM groups; (I) the secondary KEGG pathway that differed between the DM and anti_DM groups. [file Supplementary_Figures_S1.JPEG]

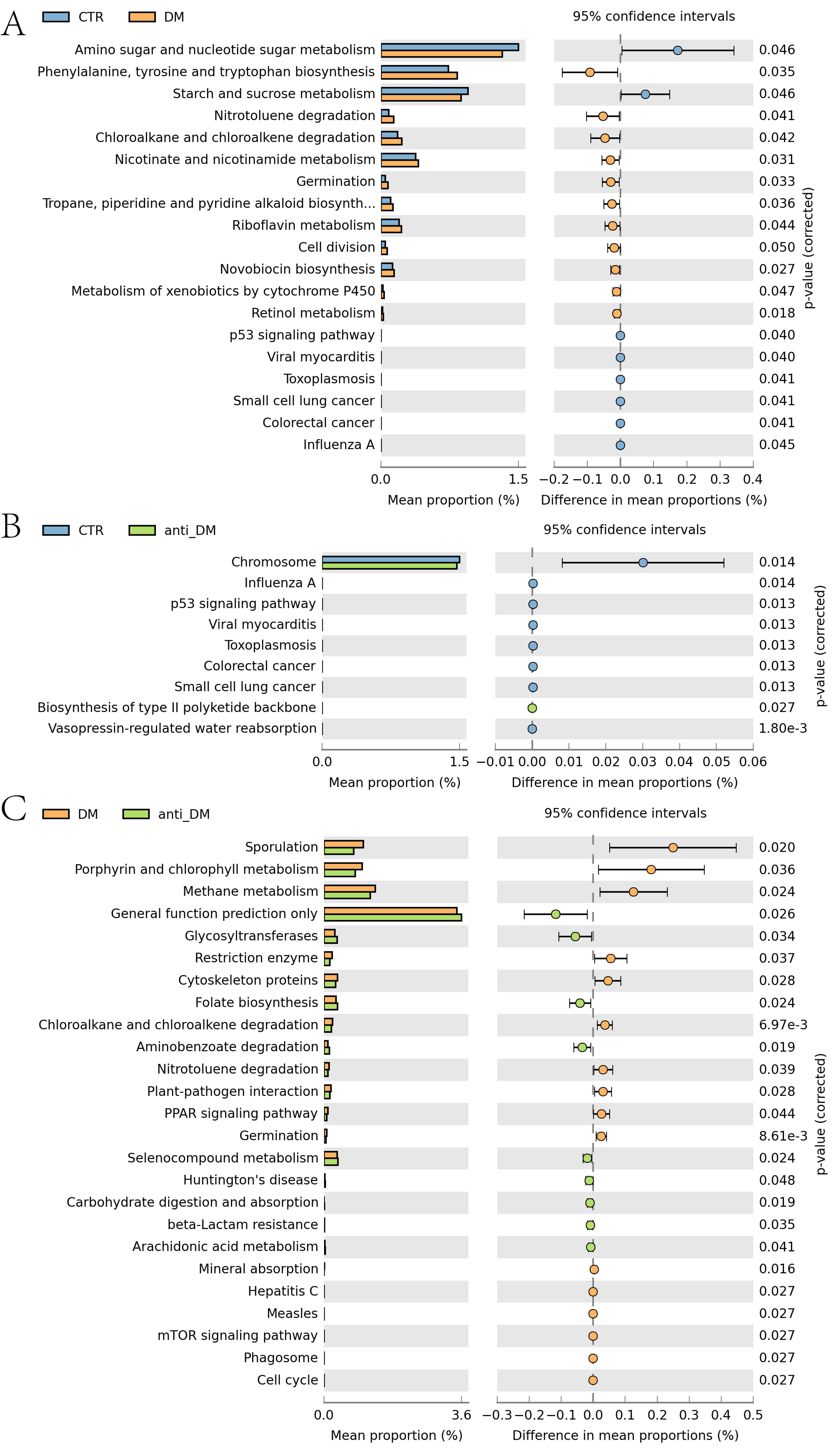

Supplement: Supplementary Figure S2 — The difference three-level KEGG pathway of 16S rRNA sequencing. (A) The three-level KEGG pathway that differed between the CTR and DM groups; (B) the three-level KEGG pathway that differed between the CTR and anti_DM groups; (C) the three-level KEGG pathway that differed between the DM and anti_DM groups. [file Supplementary_Figures_S2.JPEG]

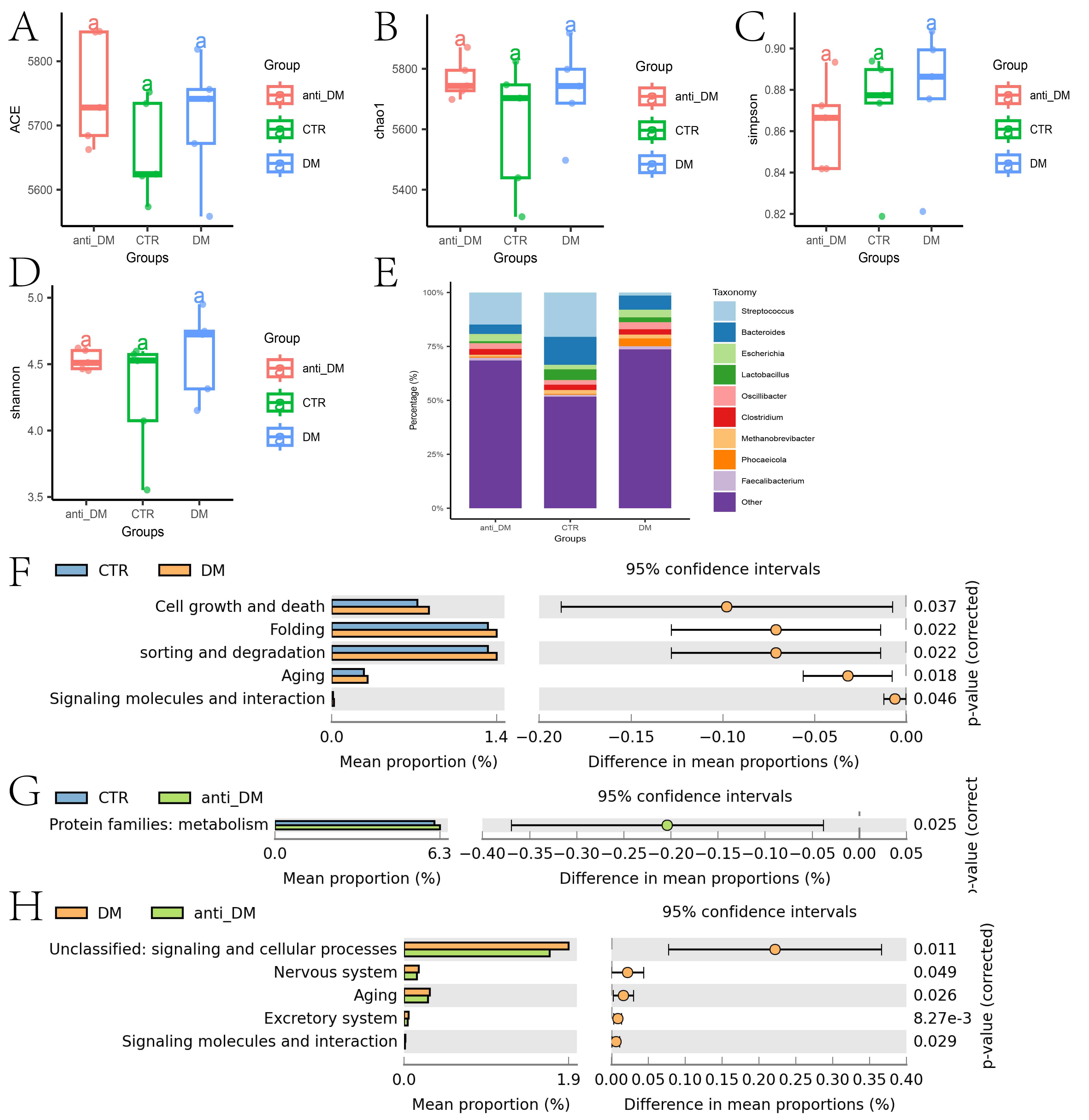

Supplement: Supplementary Figure S3 — The result of metagenome sequencing. (A) The ACE index in metagenome; (B) the Chao 1 index in metagenome; (C) the Simpson index in metagenome; (D) the Shannon index in metagenome; (E) the species composition in genus levels; F: the secondary KEGG pathway that differed between the CTR and DM groups; (G) the secondary KEGG pathway that differed between the CTR and anti_DM groups; (H) the secondary KEGG pathway that differed between the DM and anti_DM groups. [file Supplementary_Figures_S3.JPEG]

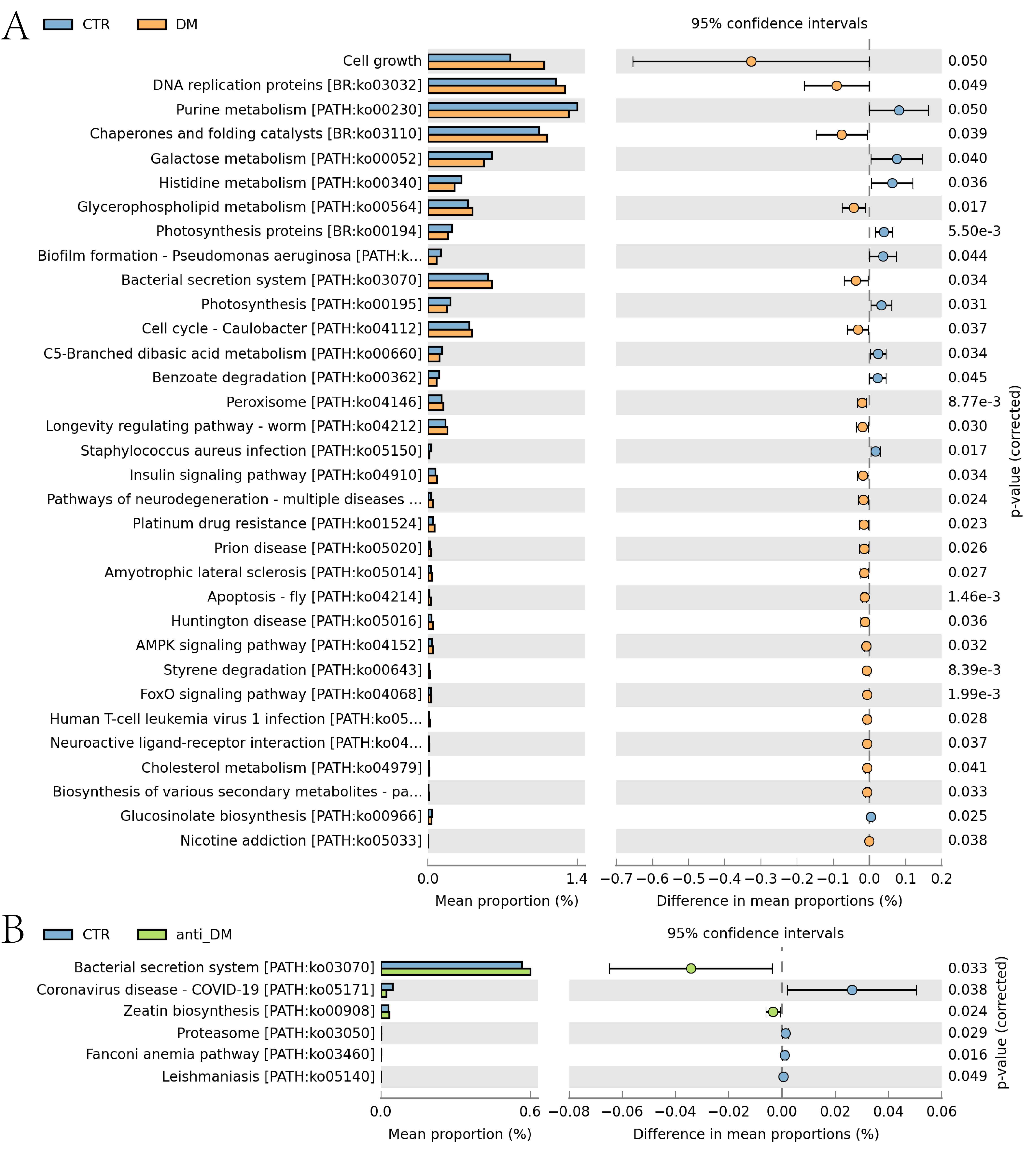

Supplement: Supplementary Figure S4 — The difference three-level KEGG pathway of metagenome sequencing. (A) The three-level KEGG pathway that differed between the CTR and DM groups; (B) the three-level KEGG pathway that differed between the CTR and anti_DM groups. [file Supplementary_Figures_S4.JPEG]

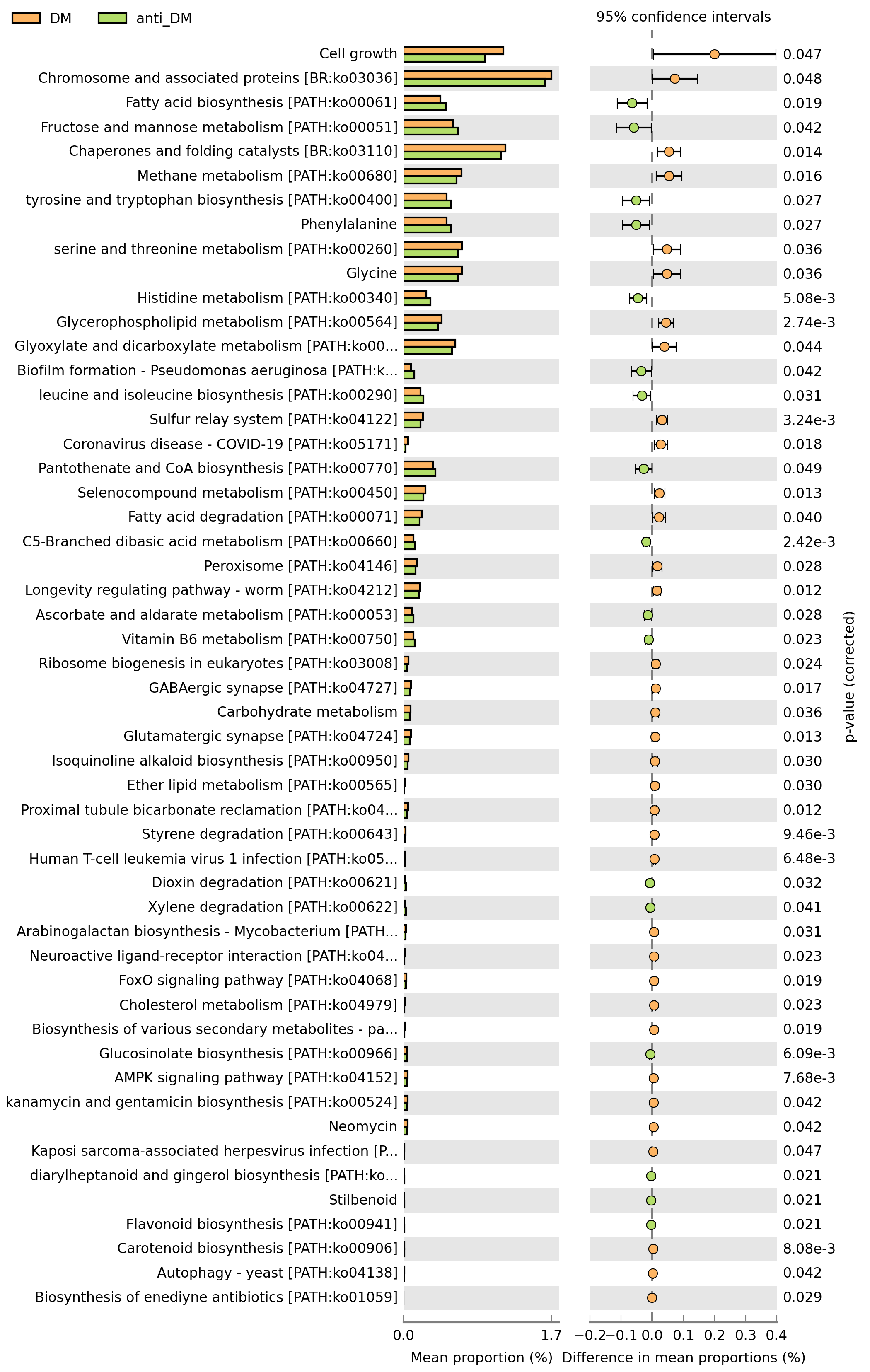

Supplement: Supplementary Figure S5 — The three-level KEGG pathway that differed between the DM and anti_DM groups. [file Supplementary_Figures_S5.JPEG]

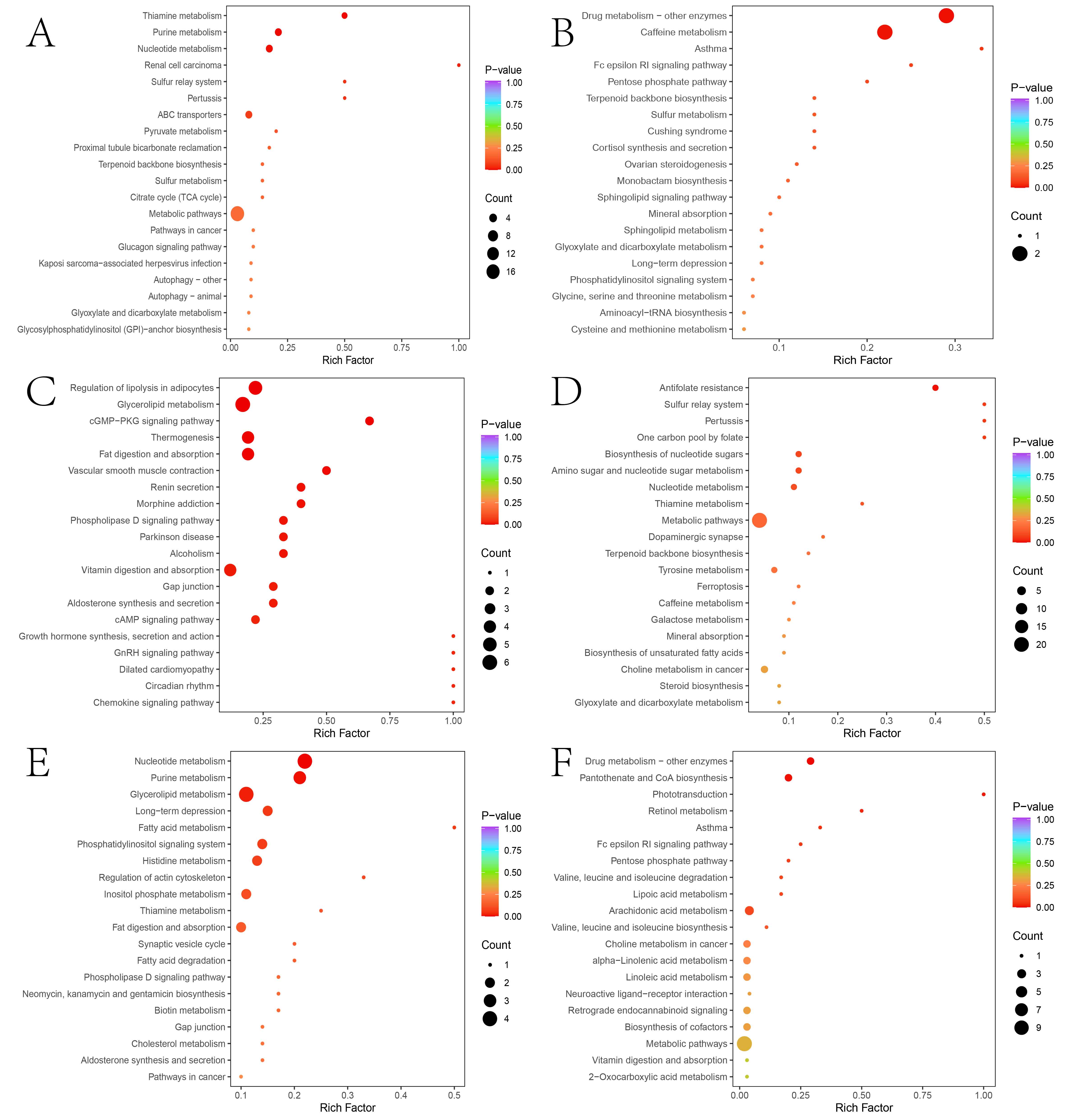

Supplement: Supplementary Figure S6 — The different metabolites of feces metabolome. (A) The significant up-regulated different metabolites between CTR group and DM group; (B) the significant down-regulated different metabolites between CTR group and DM group; (C) the significant up-regulated different KEGG pathway between CTR group and anti_DM group; (D) the significant down-regulated different KEGG pathway between CTR group and anti_DM group; (E) the significant up-regulated different metabolites between anti_DM group and DM group; (F) the significant down-regulated different metabolites between anti_DM group and DM group. [file Supplementary_Figures_S6.JPEG]
